# Exploring the neuroprotective effects of phytocannabinoids on oxygen-glucose deprived neurons in an in vitro model of stroke

**DOI:** 10.1186/s42238-026-00393-0

**Published:** 2026-01-23

**Authors:** Bhavya Chatragadda, Emily M. Potts, Alicia Collins, Hang Ma, Claudia Fallini

**Affiliations:** 1 Interdisciplinary Neuroscience Program, University of Rhode Island, Kingston, RI United States; 2 College of the Environment and Life Sciences, University of Rhode Island, Kingston, RI United States; 3 College of Pharmacy, University of Rhode Island, Kingston, RI United States; 4 Ryan Institute for Neuroscience, University of Rhode Island, Kingston, RI United States

**Keywords:** Stroke, Cannabigerorcinic acid, Oxygen-Glucose deprivation, Hypoxia, Ischemic-Reperfusion injury, iPSCs, Phytocannabinoids

## Abstract

**Background:**

Stroke is a leading cause of death and disability worldwide, but therapeutic options to reduce or prevent neuronal damage are extremely limited. Cannabinoids exhibit antioxidant, anti-inflammatory, and receptor modulatory actions that may offer neuroprotection. While research on the potential of cannabinoids has expanded in epilepsy and neurodegeneration, the neuroprotective potential of this class of natural compounds in stroke remains underexplored. Here, we evaluated a panel of phytocannabinoids (PCs) for their ability to mitigate ischemia–reperfusion injury in an in vitro human model of stroke.

**Methods:**

Human induced pluripotent stem cell (iPSC)-derived cortical neurons were subjected to 60 min of oxygen–glucose deprivation (OGD) followed by reperfusion. Neuronal survival was quantified over seven days using longitudinal live-cell imaging. Twenty-eight PCs were screened for their effect on reducing neuronal death.

**Results:**

Among 28 PCs screened, seven demonstrated modest effects, with cannabigerorcinic acid (CBGOA) significantly improving post-OGD neuronal survival. While OGD exposure led to increased cell death via activation of caspase 3, CBGOA treatment did not impact that pathway, suggesting that other caspase-independent pathways may be implicated.

**Conclusions:**

This pilot study identifies CBGOA as a candidate cannabinoid with neuroprotective potential in an in vitro model of ischemic stroke. The use of iPSC-derived human cortical neurons strengthens translational relevance, but the modest effects observed, and the limitations of in vitro systems, underscore the need for in vivo validation and further mechanistic studies. Collectively, these results provide a foundation for exploring CBGOA and related cannabinoids as potential neuroprotective agents in stroke.

**Supplementary Information:**

The online version contains supplementary material available at 10.1186/s42238-026-00393-0.

## Background

Nearly 15 million people worldwide experience a stroke each year, leaving approximately 5 million individuals permanently disabled [1]. Stroke is defined by the sudden interruption of glucose and oxygen delivery to a select region of the brain. This results from two primary forms of cerebrovascular injury: hemorrhagic, caused by rupture of a brain blood vessel, or ischemic, caused by vascular occlusion. Ischemic strokes account for more than 80% of all cases [2]. When cerebral blood flow is blocked, oxygen-glucose deprivation (OGD) triggers acute neuronal death and long-term damage to the affected brain regions. However, even after blood flow is restored through pharmacological or surgical intervention, neurons remain highly susceptible to secondary injury [3, 4]. In fact, while the rapid restoration of blood flow is crucial for improving long-term outcomes, it also initiates a second wave of neuronal death through a process known as ischemia-reperfusion injury (IRI). During ischemia, the lack of oxygen halts ATP synthesis, leading to mitochondrial damage and the disruption of cellular energy production. Upon reperfusion, the sudden influx of oxygen- and glucose-rich blood triggers oxidative stress, driven by the overproduction of free radicals and reactive oxygen species (ROS). These ROS overwhelm the cell’s natural antioxidant defenses, while already damaged mitochondria further amplify ROS production, overall exacerbating cellular dysfunction and neuronal death through the activation of the apoptotic cascade [3–6].

Due to the combined effects of these acute and sub-acute injuries, stroke survivors are at increased risk for several long-term complications, including paralysis, cognitive impairment, and emotional and behavioral changes [7–9]. Therefore, early and effective treatments to minimize neuronal death and reduce stroke-related long-term disabilities are of the utmost importance for these patients. Critically, the delayed wave of cell injury during reperfusion offers a promising window for therapeutic intervention to limit neuronal damage. To that end, antioxidants and anti-inflammatory compounds have been investigated for their potential neuroprotective or recovery-enhancing activity in post-stroke patients. Among these, derivatives from the *Cannabis sativa* plant are of great promise due to their broad-spectrum antioxidant properties and high lipophilicity [10].

More than 120 cannabinoids have been isolated and characterized from *Cannabis* [10], representing a chemically diverse class of terpenophenolic compounds distinguished by their lipophilicity and ability to readily cross the blood–brain barrier [11]. This unique chemical nature enables cannabinoids to interact with multiple molecular targets in the CNS to modulate key processes involved in neuronal survival, making them particularly promising candidates for the treatment of CNS disorders characterized by neuronal injury and/or dysregulated immune responses [12, 13]. While most research has centered on major cannabinoids such as cannabidiol (CBD) and Δ^9^-tetrahydrocannabinol (Δ^9^-THC), emerging evidence suggests that minor cannabinoids may exert distinct and sometimes stronger biological effects [14, 15]. These lesser-studied compounds often display anti-inflammatory, antioxidant, and neuroprotective actions through mechanisms beyond classical cannabinoid receptors, underscoring their potential in neuroinflammatory conditions [15–17]. Here, we evaluated an established comprehensive in-house library of structurally defined phytocannabinoids (PCs) [18] for their neuroprotective potential in an iPSC-derived cortical neuron model of ischemic stroke. This unique resource enables structure–activity relationship analyses to link specific chemical scaffolds to biological effects, advancing the discovery of novel cannabinoids capable of mitigating IRI.

## Methods

### Phytocannabinoids library

A library of structurally characterized minor phytocannabinoids (PCs) was established as described in our earlier work [18]. The library includes cannabidiolic acid methyl ester (1; CAME), Δ^9^-tetrahydrocannabutol (2; Δ^9^-THCB), cannabinol (3; CBN), cannabinodiol (4; CBND), cannabicitran (5; CBT), tetrahydrocannabinolic acid A (6; THCA-A), 11-nor-9-carboxy-Δ^9^-THC (7; THCCOOH), cannabidivarin (8; CBDV), cannabichromene (9; CBC), 11-hydroxy- Δ^9^-THC (10; 11 H- Δ^9^-THC), tetrahydrocannabivarin (11; THCV), cannabidiolic acid (12; CBDA), cannabigerovarinic acid (13; CBGVA), cannabichromevarin (14; CBCV), cannabigerovarin (15; CBGV), varinolic acid [16], cannabicyclol (17; CBL), Δ^8^-tetrahydrocannabinolic acid A (18; Δ^8^-THCA-A), cannabinol monomethyl ether (19; CBGM), cannabivarin (20; CBV), cannabigerorcinic acid (21; CBGOA), 6α-hydroxy-cannabidiol (22; 6 H-CBD), cannabigerol (23; CBG), cannabigerolic acid (24; CBGA), cannabidibutol (25; CBDB), cannabidiphorol (26; CBDP), Δ^9^-tetrahydrocannabiphorol (27; Δ^9^-THCP), and cannabidiol (28; CBD) (Supplementary Table 1).

### Cortical neuron differentiation

Human induced pluripotent stem cells (iPSCs; KOLF2.1 J, Jackson Laboratories) were maintained under standard conditions as described and differentiated into cortical neurons using the i3 method [19–21]. This approach relies on the targeted integration of a gene expression cassette into the CLYBL1 safe-harbor locus to drive the expression of the neuronal-specific transcription factor NGN2, together with an NLS-mApple fluorescent reporter and a blasticidin resistance gene (Addgene plasmid #124229) [22]. To enable integration, iPSCs were transfected with this plasmid along with Cas9 nuclease and a site-specific gRNA (Synthego). Colonies were positively selected based on blasticidin resistance (10 µg/ml) and NLS-mApple expression, pooled to minimize clonal variability, and expanded (Supplementary Fig. 1). Positivity for the stem cell marker Oct4 (Abcam, 2750) was confirmed by immunofluorescence (see below).

Differentiation was induced by adding doxycycline (2 µg/ml; Sigma Aldrich, D52071G) for 2 days, during which proliferating cells were eliminated using BrdU treatment (40µM). Cells were plated on poly-lysine (Sigma Aldrich, P7405/P3655) coated coverslips (200,000 cells/well) or 96-well plates (30,000 cells/well) and maintained in neuronal medium (Neurobasal supplemented with 2% B27, 1% N2, 1% NEAA, and 1 µg/ml laminin) for up to 14 days before exposure to ischemic injury. Successful neuronal differentiation was verified by immunostaining for MAP2 (Thermo Fisher Scientific, PA1-16751), Tau (Thermo Fisher Scientific, MN1000), GluA1 (NeuroMab, 75–327), and NR2A (NeuroMab, N327-95) (Supplementary Fig. 1). Differentiation efficiency was evaluated by scoring the number of DAPI-positive nuclei that stained positive for MAP2. Evaluation over 3 independent differentiations indicated an average efficiency of 81% (Supplementary Fig. 1).

### Ischemic injury

To model stroke in vitro, iPSC-derived neurons were subjected to 60 min of oxygen–glucose deprivation (OGD), which was selected and optimized based on established protocols [23, 24]. The complete neuronal culture medium was removed and replaced with glucose-free Neurobasal medium, and cells were immediately transferred to a hypoxia chamber (1% O₂, 37 °C) for 60 min. Following OGD, cultures were returned to normoxic conditions, and the glucose-free medium was replaced with one of the following: (i) complete neuronal medium with DMSO (*OGD + DMSO*), (ii) complete neuronal medium containing test PCs at the desired concentrations (*OGD + PC*), or (iii) complete neuronal medium supplemented with 25 mM sodium arsenite to induce rapid cell death. To control for the possible cell death caused by cell handling, control neurons underwent similar media changes as OGD neurons but were kept under normal oxygen and glucose concentration for the duration of the experiment (*Ctrl*).

### Compound screening

The panel of 28 PCs was screened for their ability to enhance neuronal survival relative to OGD-DMSO controls. Each compound was randomly assigned an identification number (i.e., 1 to 28) and these IDs were used consistently across all experiments (Supplementary Table 1). After a 60-minute OGD, cells grown in 96-well plates were treated with each compound at a final concentration of 1 µM in triplicates and monitored for survival over seven days. A subset of seven compounds was further evaluated across a dose range (0.25, 0.5, 1, 2, and 4 µM) using the same OGD paradigm and imaging protocol described above. An untreated control (i.e. OGD-DMSO) and negative control (i.e., sodium arsenite) were always included. The distribution of compounds across the plate was randomized for each biological replicate. To assess neuronal survival, cells were imaged in the IncuCyte system (S3, Sartorius) at 20x magnification in both phase contrast and red fluorescence channels to detect mApple expression, used as a marker of viability. Raw 32-bit red fluorescence images were exported and processed in Fiji/ImageJ [25] using ROF denoise (theta = 25), top-hat (radius = 20 pixels), despeckle, and smooth filters, followed by thresholding and particle analysis (Supplementary Fig. 2). Live neuron counts were quantified as the number of red fluorescent bodies per image. A lower limit of 30 pxls^2^ was used to remove artifacts and ensure only live cells were counted. To account for variability across experiments, counts at each time point were normalized to the corresponding baseline (i.e., day 0).

### Apoptosis assay

To evaluate the activation of the apoptotic pathway, neurons were cultured on glass coverslips (15 mm) and exposed to OGD ± compound as described above. Neurons were then fixed in 4% paraformaldehyde for 15 min and processed for immunofluorescence as described [19, 20]. Fixation and staining were performed at 0, 1, and 7 days post-treatment. Primary antibodies were incubated at 4 °C overnight and included anti–cleaved caspase 3 (Cell Signaling – 9661) and anti-MAP2 (Thermo Fisher Scientific - PA1-16751), and anti-GluA1 (NeuroMab – 75–327). Species-specific secondary antibodies conjugated with Alexa Fluor 488 and 647 dyes (Jackson Immunoresearch) were incubated at room temperature for 1 h.

Coverslips were imaged at 20x magnification using a Leica DMi8 Thunder widefield microscope equipped with a cooled cMOS camera (DFC9000 GTC, Leica) and analyzed using Fiji/ImageJ in blind. To quantify cleaved caspase 3 fluorescence intensity, the fluorescent signal was thresholded with a fixed threshold across experimental conditions and the background was cleared. Regions of interest (ROIs) were defined based on DAPI staining to identify cell bodies, and cleaved caspase 3 fluorescence intensity was measured within these ROIs.

### Statistical analyses

Normality of data was assessed with the Shapiro-Wilk test for small dataset. Data where points were matched between the groups (averaged biological replicates) were analyzed by ratio paired *t* tests. For experiments involving multiple treatment groups relative to a common untreated control, mixed-effects one-way or two-way ANOVAs were used, followed by Dunnett’s multiple comparisons post *hoc* test to correct for multiple testing. Nonlinear regression analysis of survival time course was performed using one-phase decay function, defined as

$$y=({y}_{t0}-Plateau){e}^{-kx}$$

where *y*_*t0*_ represents neuronal survival at time 0, *Plateau* represents y value at infinite times, and *k* is the decay constant expressed as *days*^*− 1*^, with higher values indicating slower decay. The decay constant *k* was calculated for each biological replicate and compared between DMSO and PC-treated samples. All analyses were performed in GraphPad Prism 9.0. *P* values < 0.05 were considered significant. Effect sizes (*d*) were calculated by dividing the mean of differences by the standard deviation of differences for paired t tests, or by the residuals standard deviation for mixed effects analyses. Given the small sample sizes, d was corrected using the factor J (for *n* = 4, J = 0.7236; for *n* = 5, J = 0.7833). Effect sizes and 95% confidence intervals (CI) were reported in the figure legends. Independent biological replicates are shown as separate data points.

### Experimental reproducibility

All experiments were repeated across at least 3 independent iPSC differentiations, with technical replicates included for each condition. For survival assays, 3–5 wells (i.e. technical replicates) were averaged per condition per biological replicate (i.e. independent neuronal differentiation). For cleaved caspase 3 assays, a minimum of 100 cells were quantified per condition and averaged per biological replicate. All statistical analyses were performed only considering data from biological replicates. The number of independent replicates per experiment is specified in each figure legend as *n*.

## Results

### OGD causes neuronal death

**Fig. 1:**
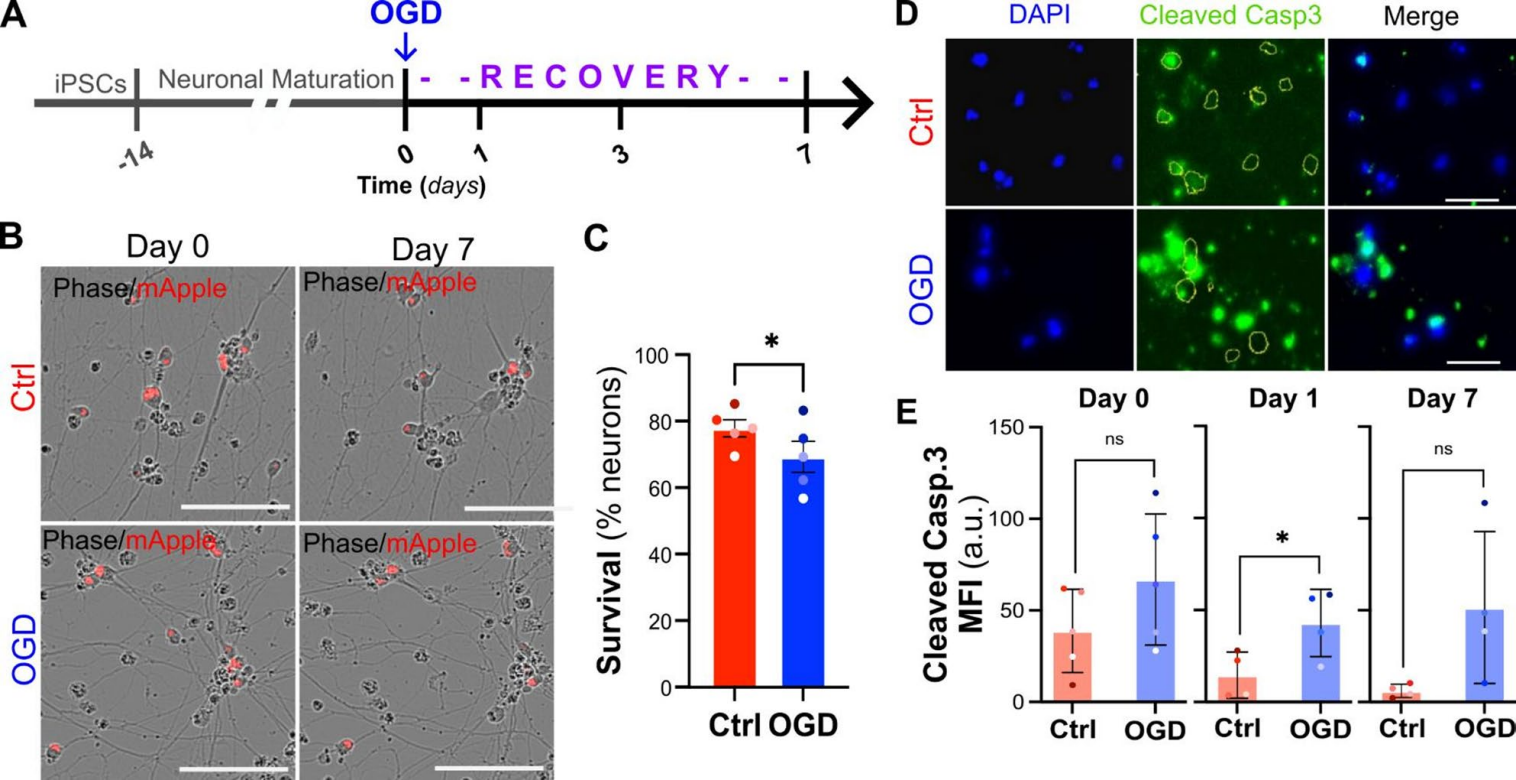
OGD reduces neuronal survival and induces caspase 3 activation in iCNs. **A**. Schematics of the experimental timeline. Two-week-old human iCNs were subjected to 1 h of oxygen-glucose deprivation (OGD), followed by a 7-day recovery period. **B**. Representative phase contrast and mApple fluorescence images of control (Ctrl) or OGD-exposed iCNs at Day 0 and Day 7 following stress. **C**. Quantification of neuronal survival at day 7 shows an 8% reduction in survival in OGD-treated neurons compared with CTRL (77.9 ± 2.6 Ctrl vs. 69.3 ± 4.6 OGD, paired two-tailed t test, *n* = 5, *p* = 0.0183, d = 1.35, CI = -14.77 to -2.389). **D**. Representative immunofluorescence images of cleaved caspase 3 (green) and DAPI (blue) in Ctrl and OGD neurons at recover day 1. **E**. Quantification of cleaved caspase 3 mean fluorescence intensity (MFI) at recovery day 0, 1, and 7. Caspase 3 activation was significantly increased on day 1 in OGD neurons relative to Ctrl (14.56 ± 6.24 Ctrl vs. 42.92 ± 9.13 OGD, ratio paired two-tailed t test, *n* = 4, *p* = 0.0315, d = 1.38, CI = 1.261 to 12.51), but not at Day 0 or Day 7. Data are shown as mean ± SEM. Scale bars: 100 μm in B, 50 μm in D

To establish an in vitro model of stroke, we exposed 2-week-old iPSC-derived cortical neurons (iCNs) to 60-minute oxygen–glucose deprivation (OGD) and tracked their survival every 24 h over a period of 7 days. As control for the accuracy of the analysis pipeline, neurons were treated with a high dose of sodium arsenite, which led to a quick and dramatic increase in cell death (Supplementary Fig. 2). We found that OGD-exposed neurons showed a significantly lower survival rate 7 days after the initial injury when compared to control neurons that were maintained under normoxic conditions (69.2% OGD vs. 77.7% Ctrl survival, Fig. 1A-C). To investigate whether apoptosis contributes to OGD-induced neuronal death, we quantified the levels of cleaved (i.e., active) caspase 3 expression in control and OGD neurons at day 0, day 1, and day 7. OGD neurons displayed a significant increase in cleaved caspase 3 expression on day 1 but not day 7 (Fig. 1D), suggesting that the activation of the apoptotic cascade is an early but transient event following ischemic stroke in vitro. Overall, these data support the use of this stress paradigm to assess the potential of cannabinoids to modulate neuronal toxicity after stroke.

### Cannabinoid treatment reduces neuronal death in a concentration-dependent manner

**Fig. 2:**
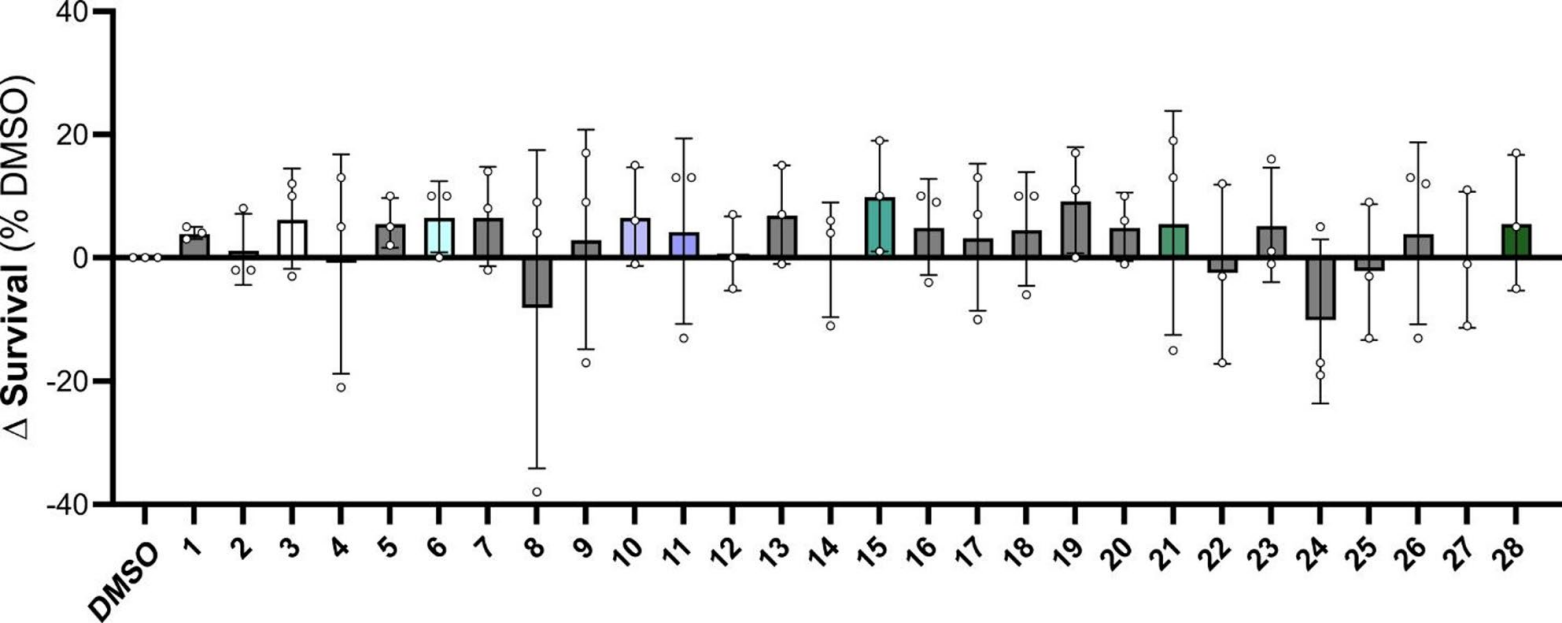
Screening of phytocannabinoids identifies candidates that modulate neuronal survival after OGD. iPSC-derived cortical neurons were subjected to OGD and subsequently treated with a panel of 28 PCs (1 µM) or vehicle control (DMSO). Quantification of neuronal survival on day 7 identified a subset of samples (3, 6, 10, 11, 15, 21, and 28) with mild protective effects. Data were normalized to the DMSO control to correct for inter-experimental variability (see Supplementary Fig. 3 for raw values). Data are presented as mean ± SEM, with individual biological replicates shown (*n* = 3, one-way ANOVA with mixed effects model, not significant)

After confirming that OGD significantly decreases neuronal survival, we screened a panel of 28 phytocannabinoids (PCs) on post-OGD neurons to assess their potential neuroprotective effect. Neurons were treated with each compound (1 µM) immediately after OGD and survival was monitored for up to 7 days. DMSO was used as vehicle control. Under these conditions, we found that several compounds had no obvious effect on neuronal survival compared to DMSO-treated controls at day 7 (1, 2, 4, 5, 7, 9, 13, 16, 17, 18, 19, 20, 23, 26, and 27), or mildly increased toxicity (8, 22, 24, and 25). However, a subset of compounds demonstrated a mild protective effect, even though it did not reach statistical significance (3, 6, 10, 11, 15, 21, and 28; Fig. 2 and Supplementary Fig. 3).

**Fig. 3:**
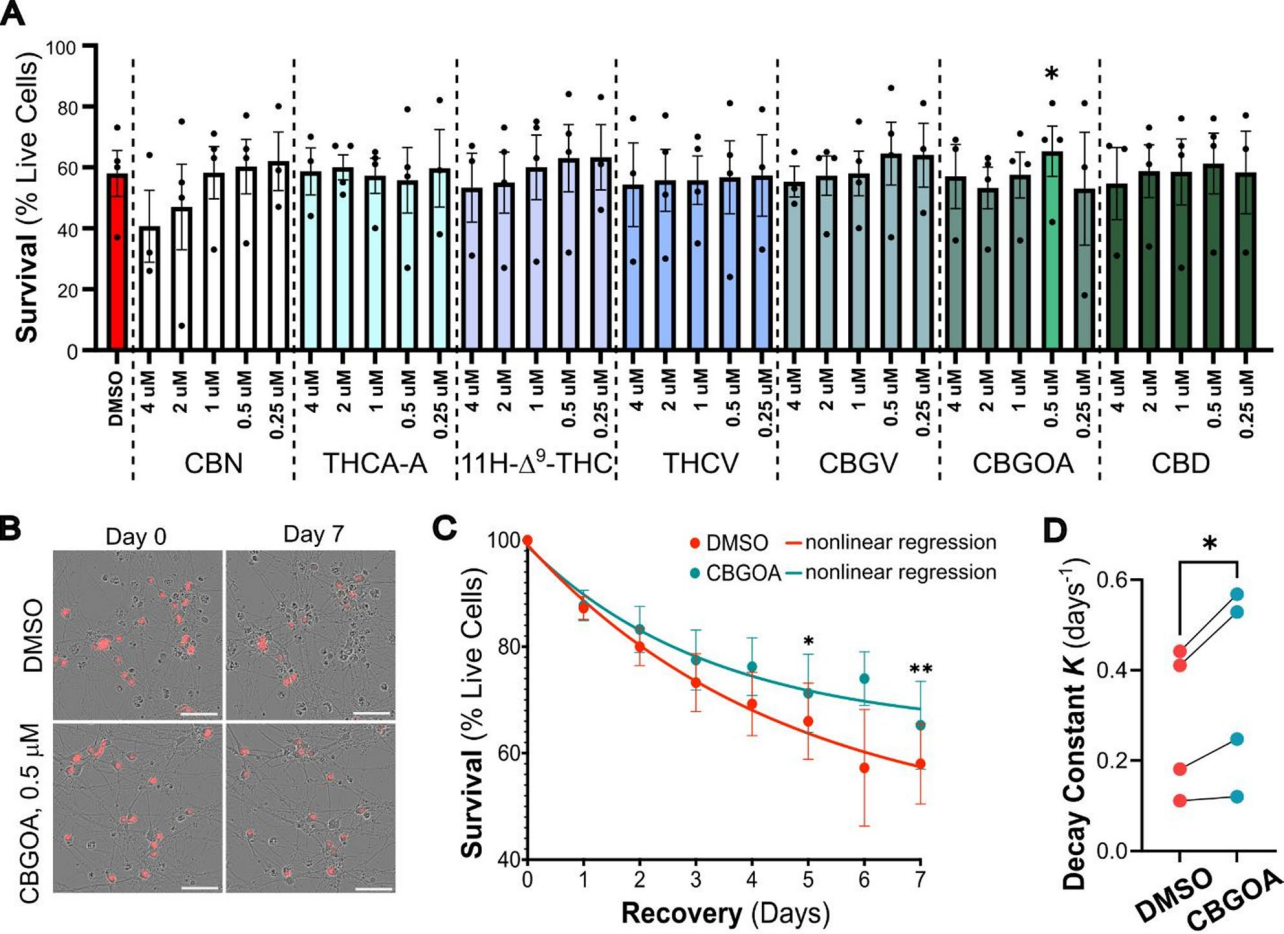
CBGOA improves survival after OGD in concentration-response testing. **A** Survival of iCNs exposed to OGD and treated with decreasing concentration of phytocannabinoids (survival is shown as percentage of initial cell numbers). Cells were treated with each compound at 4, 2, 1, 0.5, and 0.25 µM, and survival was measured after 7 days. CBGOA (0.5 µM) significantly increased neuronal survival compared to DMSO-treated controls (mixed-effects ANOVA with Dunnett’s correction for multiple comparisons (DMSO vs. PC), *n* = 4, *p* = 0.0136, d = 0.9, CI = -11.80 to -2.699). Data are shown as mean ± SEM, with individual biological replicates indicated. **B** Representative phase contrast and mApple fluorescence images of DMSO- and CBGOA-treated neurons at day 0 and day 7. Scale bar, 50 μm. **C** Time-course analysis of neuronal survival across 7 days of recovery shows prolonged preservation of viability with CBGOA treatment compared to DMSO alone (multiple paired t test with Bonferroni-Dunn correction, *n* = 4, *p* = 0.028 at day 5, *p* = 0.005 at day 7). Nonlinear regression analysis is superimposed to the experimental data. **D** Decay constant K, extrapolated from regression analysis, shows a significant increase in CBGOA-treated iCNs, indicating slower decay and longer survival (mean increase from 0.28 to 0.36 days^− 1^, ratio paired t test, *n* = 4, *p* = 0.0315, d = 1.58, CI = 1.063 to 1.472)

Based on these observations, we wondered whether the effects of these compounds could be enhanced by either increasing or decreasing their concentration. Thus, we selected a smaller panel of compounds that had shown some effect in at least two of the three trials: 3 (CBN), 6 (THCA-A), 10 (11 H-Δ^9^-THC), 11 (THCV), 15 (CBGV), 21 (CBGOA), and 28 (CBD). Neurons were treated with each compound at five decreasing concentrations (4, 2, 1, 0.5, and 0.25 µM) immediately after OGD, and their survival was monitored as described above over the course of 7 days (Fig. 3). Not all compounds showed consistent dose dependence, but overall lower concentrations (0.25–0.5 µM) tended to perform as well or better than the 1 µM dose used in the initial screen. Among these, 0.5 µM cannabigerorcinic acid (CBGOA, compound 21) significantly increased neuronal survival compared to OGD-DMSO controls (65.3% versus 58.0%; Fig. 3).

To assess whether CBGOA treatment rescued neuronal survival via the downregulation of the apoptotic pathway, we quantified caspase 3 activation in neurons exposed to OGD and treated with CBGOA or DMSO only. Surprisingly, we found that CBGOA treatment did not impact cleaved caspase 3 levels in OGD-exposed neurons relative to controls (Supplementary Fig. 4), suggesting that CBGOA may mitigate OGD-induced neuronal death through alternative pathways.

## Discussion

Stroke is a leading cause of death and long-term disability in the US and globally, and yet therapeutic options that can lessen the neuronal damage caused by the acute ischemic and subacute reperfusion injuries are limited. In this study, we assessed the potential of phytocannabinoids (PCs) to promote survival of iPSC-derived cortical neurons (iCNs) exposed to 60 min oxygen-glucose deprivation (OGD).

Our findings indicate that among a panel of 28 PCs tested in our in vitro model, CBGOA demonstrated a modest but significant neuroprotective effect, increasing neuronal survival after OGD by 7.25% compared to DMSO-treated neurons. While CBGOA was the only compound to achieve a statistically significant effect, other phytocannabinoids (i.e., CBN, THCA-A, 11 H-Δ^9^-THC, THCV, CBGV, and CBD) demonstrated mild protective effects. Interestingly, this subset of compounds shares notable structural similarities that may underlie their biological activity. These cannabinoids largely preserve the core dibenzopyran or olivetol-derived backbone characteristic of phytocannabinoids, while varying in side-chain length, degree of oxidation, and the presence of acidic or neutral functional moieties. Preliminary structure–activity relationship observations suggest that minor modifications on the resorcinol ring and the length or branching of the alkyl side chain can markedly influence neuroprotective efficacy. For instance, the enhanced activity of CBGOA relative to its biosynthetic precursors (CBG and CBGA) may derive from the presence of the olivetolic acid moiety, which could improve receptor interaction [26]. Similarly, the mild but relatively consistent protection observed among CBN, THCV, and 11 H-Δ^9^-THC analogs implies that partial oxidation and short alkyl substitutions do not abolish activity but may modulate target affinity or lipid membrane interaction [27].

CBGOA is a precursor in the biosynthesis of Δ^9^-THC, which is the principal psychoactive component of *Cannabis*. While limited studies are available on the cellular effects of this PC, it was recently shown to have anticonvulsant effects in a Scn1a^+/−^ mouse model of Dravet syndrome, implicating interactions with GPR55, TRPV1 channels, and GABA_*A*_ receptors [28]. While further experimental evidence is needed to assess the relevance of these pathways to PCs activity in stroke, previous studies have shown their involvement in mediating excitotoxicity [29], calcium dysregulation [30], and neuronal death [31], processes that are activated in ischemic tissues. While the precise mechanism of action of CBGOA in iCNs remains unclear, its distinct pharmacological profile compared to more abundant cannabinoids such as CBD or Δ^9^-THC suggests that it may act through complementary or novel pathways. It is noteworthy that CBGOA’s chemical stability likely plays a critical role in its bioavailability and therapeutic efficacy. As an acidic cannabinoid, it is susceptible to decarboxylation and oxidation, which may limit its translational potential. Further studies, including evaluation of its stability and pharmacokinetics in animal models, are warranted to advance cannabinoid-based therapeutics for IRI. Nevertheless, the findings from this pilot study add to this emerging evidence, positioning CBGOA as a candidate for further mechanistic and translational studies.

Cannabinoid research has gained considerable momentum in recent years, with accumulating evidence supporting their therapeutic potential in neurological disorders such as epilepsy [32], Alzheimer’s disease [33], and Parkinson’s disease [34]. In contrast, their role in stroke pathophysiology and recovery remains underexplored. Despite decades of research, no neuroprotective agents have been approved for stroke, leaving a critical therapeutic gap. Cannabinoids, with their combined antioxidant, anti-inflammatory, and receptor-modulating actions, may be uniquely positioned to address this unmet need [35]. Our results build on prior work showing that cannabinoids can influence neuronal survival and excitotoxicity [33–36]. We extend these findings by identifying CBGOA as a modest but significant modulator of neuronal survival and a potential candidate for future in vivo studies for post-stroke neuroprotection.

### Limitations

An important strength of this study is the use of iPSC-derived human cortical neurons, which provides a translationally relevant platform compared to rodent primary cultures. This model enables the evaluation of human-specific neuronal responses and improves the potential applicability of findings to human disease [37, 38]. However, it is important to note that in vitro systems cannot fully recapitulate the cellular complexity of the brain or the systemic factors involved in stroke injury and recovery, including vascular and immune responses to the injury [39, 40]. The observed effects, while encouraging, are modest and cannot be assumed to translate to in vivo efficacy or clinical benefit without further validation. Comprehensive follow-up studies, including in vivo stroke models, expanded dose-response testing, and mechanistic validation, will be essential before any assessment of clinical relevance can be made. Future studies including known neuroprotective compounds of clinical relevance would also be helpful to evaluate the magnitude of the pro-survival effect of PCs.

Finally, safety and pharmacological considerations must be acknowledged. Phytocannabinoids vary in their psychoactive and physiological effects, and CBGOA’s safety profile has not been extensively characterized. Future investigations will therefore need to consider pharmacokinetics, tolerability, and off-target effects alongside efficacy.

## Conclusions

This study provides new evidence supporting the neuroprotective effect of the phytocannabinoid CBGOA in an in vitro model of stroke. While future studies should be carried out to dissect the molecular targets and cellular pathways modulated by CBGOA, the data presented in this brief report suggest this class of compounds may represent a novel tool to reduce neuronal damage following brain ischemia.

## Supplementary Information

Supplementary Material 1.

## Data Availability

The data generated through this research are available from the corresponding authors upon reasonable request.

## Abbreviations

OGD: Oxygen-glucose deprivation
IRI: Ischemia-reperfusion injury
ROS: Reactive Oxygen Species
PC: Phytocannabinoids
iPSC: Induced pluripotent stem cell
iCN: iPSC-derived cortical neuron
CBGOA: cannabigerorcinic acid

## References

[1] Feigin VL, Brainin M, Norrving B, Martins SO, Pandian J, Lindsay P, et al. World stroke organization: global stroke fact sheet 2025. Int J Stroke. 2025;20(2):132–44. 39635884 10.1177/17474930241308142 PMC11786524

[2] Renedo D, Acosta JN, Leasure AC, Sharma R, Krumholz HM, de Havenon A, et al. Burden of ischemic and hemorrhagic stroke across the US from 1990 to 2019. JAMA Neurol. 2024;81(4):394–404. 38436973 10.1001/jamaneurol.2024.0190 PMC10913004

[3] Stroke Reperfusion ARKMDVJ. Injury. 2025. Available from: https://www.ncbi.nlm.nih.gov/books/NBK564350/.

[4] P C, R F. Pathophysiology of Reperfusion Injury. In 2011. Available from: https://www.ncbi.nlm.nih.gov/books/NBK534267/. 30485021

[5] Kalogeris T, Baines C, Krenz M, Korthuis R. Cell biology of ischemia/reperfusion injury. Int Rev Cell Mol Biology. 2012;298:229–317. 10.1016/B978-0-12-394309-5.00006-7 PMC3904795 22878108

[6] Kalaria RN. The pathology and pathophysiology of vascular dementia. Neuropharmacology. 2018;134:226–39. 29273521 10.1016/j.neuropharm.2017.12.030

[7] Chohan S, Venkatesh P, How C. Long-term complications of stroke and secondary prevention. Singap Méd J. 2019;60:616–20. 10.11622/smedj.2019158 PMC7911065 31889205

[8] Rost NS, Brodtmann A, Pase MP, van Veluw SJ, Biffi A, Duering M, et al. Post-Stroke cognitive impairment and dementia. Circ Res. 2022;130:1252–71. 35420911 10.1161/CIRCRESAHA.122.319951

[9] Kim JS. Post-stroke mood and emotional disturbances. J Stroke. 2016;18:244–55. 27733031 10.5853/jos.2016.01144 PMC5066431

[10] Al-Khazaleh AK, Zhou X, Bhuyan DJ, Münch GW, Al-Dalabeeh EA, Jaye K, et al. The neurotherapeutic arsenal in cannabis sativa. Molecules. 2024;29:410. 38257323 10.3390/molecules29020410 PMC10821245

[11] Calapai F, Cardia L, Sorbara EE, Navarra M, Gangemi S, Calapai G, et al. Cannabinoids, Blood–Brain Barrier, and brain disposition. Pharmaceutics. 2020;12(3):265. 32183416 10.3390/pharmaceutics12030265 PMC7150944

[12] Brook E, Mamo J, Wong R, Al-Salami H, Falasca M, Lam V, et al. Blood-brain barrier disturbances in diabetes-associated dementia: therapeutic potential for cannabinoids. Pharmacol Res. 2019;141:291–7. 30616019 10.1016/j.phrs.2019.01.009

[13] Sánchez AJ, García-Merino A. Neuroprotective agents: cannabinoids. Clin Immunol. 2012;142(1):57–67. 21420365 10.1016/j.clim.2011.02.010

[14] Stone NL, Murphy AJ, England TJ, O’Sullivan SE. A systematic review of minor phytocannabinoids with promising neuroprotective potential. Br J Pharmacol. 2020;177:4330–52. 32608035 10.1111/bph.15185 PMC7484504

[15] Jurga M, Jurga A, Jurga K, Kaźmierczak B, Kuśmierczyk K, Chabowski M. Cannabis-Based phytocannabinoids: Overview, mechanism of Action, therapeutic Application, Production, and affecting environmental factors. Int J Mol Sci. 2024;25:11258. 39457041 10.3390/ijms252011258 PMC11508795

[16] Bhunia S, Kolishetti N, Arias AY, Vashist A, Nair M. Cannabidiol for neurodegenerative disorders: A comprehensive review. Front Pharmacol. 2022;13:989717. 36386183 10.3389/fphar.2022.989717 PMC9640911

[17] Tomaszewska-Zaremba D, Gajewska A, Misztal T. Anti-Inflammatory effects of cannabinoids in therapy of neurodegenerative disorders and inflammatory diseases of the CNS. Int J Mol Sci. 2025;26:6570. 40724820 10.3390/ijms26146570 PMC12294762

[18] Liu C, Puopolo T, Li H, Cai A, Seeram NP, Ma H. Identification of SARS-CoV-2 main protease inhibitors from a library of minor cannabinoids by biochemical Inhibition assay and surface plasmon resonance characterized binding affinity. Molecules. 2022;27(18):6127. 36144858 10.3390/molecules27186127 PMC9502466

[19] Sirtori R, J Gregoire M, Potts M, Collins E, Donatelli A, Fallini L. LINC complex alterations are a key feature of sporadic and Familial ALS/FTD. Acta Neuropathol Commun. 2024;12(1):69. 38664831 10.1186/s40478-024-01778-z PMC11046770

[20] Jean Gregoire M, Sirtori R, Donatelli L, Morgan Potts E, Collins A, Zamor D, et al. Early disruption of the CREB pathway drives dendritic morphological alterations in FTD/ALS cortical neurons. Proc Natl Acad Sci U S A. 2024;121(49):e2406998121. 39589881 10.1073/pnas.2406998121 PMC11626127

[21] Fernandopulle MS, Prestil R, Grunseich C, Wang C, Gan L, Ward ME. Transcription Factor-Mediated differentiation of human iPSCs into Neurons. Current protocols in cell biology / editorial board. Juan S Bonifacino. 2018;79(1):e51. 10.1002/cpcb.51 PMC6993937 29924488

[22] Tian R, Gachechiladze MA, Ludwig CH, Laurie MT, Hong JY, Nathaniel D, et al. CRISPR Interference-Based platform for multimodal genetic screens in human iPSC-Derived neurons. Neuron. 2019;104(2):239–e25512. 31422865 10.1016/j.neuron.2019.07.014 PMC6813890

[23] Tasca CI, Dal-Cim T, Cimarosti H. Neuronal cell Death, methods and protocols. Methods Mol Biol. 2014;1254:197–210. 10.1007/978-1-4939-2152-2_15 25431067

[24] Goldberg M, Choi D. Combined oxygen and glucose deprivation in cortical cell culture. J Neurosci. 1993;13:3510–24. 8101871 10.1523/JNEUROSCI.13-08-03510.1993 PMC6576549

[25] Schindelin J, Arganda-Carreras I, Frise E, Kaynig V, Longair M, Pietzsch T, et al. Fiji: an open-source platform for biological-image analysis. Nat Methods. 2012;9(7):676–82. 22743772 10.1038/nmeth.2019 PMC3855844

[26] Reggio PH. Endocannabinoid binding to the cannabinoid receptors: what is known and what remains unknown. Curr Med Chem. 2010;17(14):1468–86. 10.2174/092986710790980005. 10.2174/092986710790980005 PMC4120766 20166921

[27] Alhamoruni A, Lee AC, Wright KL, Larvin M, O’Sullivan SE. Pharmacological effects of cannabinoids on the Caco-2 cell culture model of intestinal permeability. J Pharmacol Exp Ther. 2010;335(1):92–102. 20592049 10.1124/jpet.110.168237

[28] Anderson LL, Udoh M, Everett-Morgan D, Heblinski M, McGregor IS, Banister SD, et al. Olivetolic acid, a cannabinoid precursor in cannabis sativa. J Cannabis Res. 2022;4:2. 34980287 10.1186/s42238-021-00113-w PMC8725448

[29] Dong X xia, Wang Y, Qin Zzhong. Molecular mechanisms of excitotoxicity and their relevance to pathogenesis of neurodegenerative diseases. Acta Pharmacol Sin. 2009;30:379–87. 19343058 10.1038/aps.2009.24 PMC4002277

[30] Kristián T, Siesjö BK. Calcium in ischemic cell death. Stroke. 1998;29:705–18. 9506616 10.1161/01.str.29.3.705

[31] Kasatkina LA, Rittchen S, Sturm EM. Neuroprotective and Immunomodulatory action of the endocannabinoid system under neuroinflammation. Int J Mol Sci. 2021;22:5431. 34063947 10.3390/ijms22115431 PMC8196612

[32] Perucca E. Cannabinoids in the treatment of epilepsy: hard evidence at last? J Epilepsy Res. 2017;7:61–76. 29344464 10.14581/jer.17012 PMC5767492

[33] Aso E, Ferrer I. Cannabinoids for treatment of alzheimer’s disease: moving toward the clinic. Front Pharmacol. 2014;5:37. 24634659 10.3389/fphar.2014.00037 PMC3942876

[34] Fernández-Ruiz J, Sagredo O, Pazos MR, García C, Pertwee R, Mechoulam R, et al. Cannabidiol for neurodegenerative disorders. Br J Clin Pharmacol. 2013;75:323–33. 22625422 10.1111/j.1365-2125.2012.04341.x PMC3579248

[35] Atalay S, Jarocka-Karpowicz I, Skrzydlewska E. Antioxidative and Anti-Inflammatory properties of Cannabidiol. Antioxidants. 2019;9:21. 31881765 10.3390/antiox9010021 PMC7023045

[36] Marsicano G, Goodenough S, Monory K, Hermann H, Eder M, Cannich A, et al. CB1 cannabinoid receptors and On-Demand defense against excitotoxicity. Science. 2003;302:84–8. 14526074 10.1126/science.1088208

[37] Shi Y, Kirwan P, Livesey FJ. Directed differentiation of human pluripotent stem cells to cerebral cortex neurons and neural networks. Nat Protoc. 2012;7:1836–46. 22976355 10.1038/nprot.2012.116

[38] Kelava I, Lancaster MA. Stem cell models of human brain development. Cell Stem Cell. 2016;18:736–48. 27257762 10.1016/j.stem.2016.05.022

[39] Mergenthaler P, Dirnagl U, Meisel A. Pathophysiology of stroke: lessons from animal models. Metab Brain Dis. 2004;19:151–67. 15554412 10.1023/b:mebr.0000043966.46964.e6

[40] Fisher M, Feuerstein G, Howells DW, Hurn PD, Kent TA, Savitz SI, et al. Update of the stroke therapy academic industry roundtable preclinical recommendations. Stroke. 2009;40:2244–50. 19246690 10.1161/STROKEAHA.108.541128 PMC2888275

