# Supplementary material for: Exploring the neuroprotective effects of phytocannabinoids on oxygen-glucose deprived neurons in an in vitro model of stroke
Source: J Cannabis Res. 2026 Jan 23;8:29. doi: 10.1186/s42238-026-00393-0 (PMC12911164; doi:10.1186/s42238-026-00393-0)
Supplement: Supplementary file 1 — Supplementary Material 1. [file 42238_2026_393_MOESM1_ESM.docx]

Supplementary Information for:

Exploring the neuroprotective effects of phytocannabinoids on oxygen-glucose deprived neurons in an *in-vitro* model of stroke

Bhavya Chatragadda*, Emily M. Potts^*^, Alicia Collins, Hang Ma, Claudia Fallini

Content:

**Supplementary Figure 1.** *Characterization of Kolf2.1J iPSC-derived cortical neurons.*

**Supplementary Figure 2.** *Validation of live-cell screening pipeline using sodium arsenite (NaArs) as toxic agent.*

**Supplementary Figure 3.** *Phytocannabinoids modulate neuronal survival after OGD.*

**Supplementary Figure 4.** *CBGOA treatment does not affect caspase-3 activation.*

**Supplementary Table 1.** *List of the tested phytocannabinoids.*


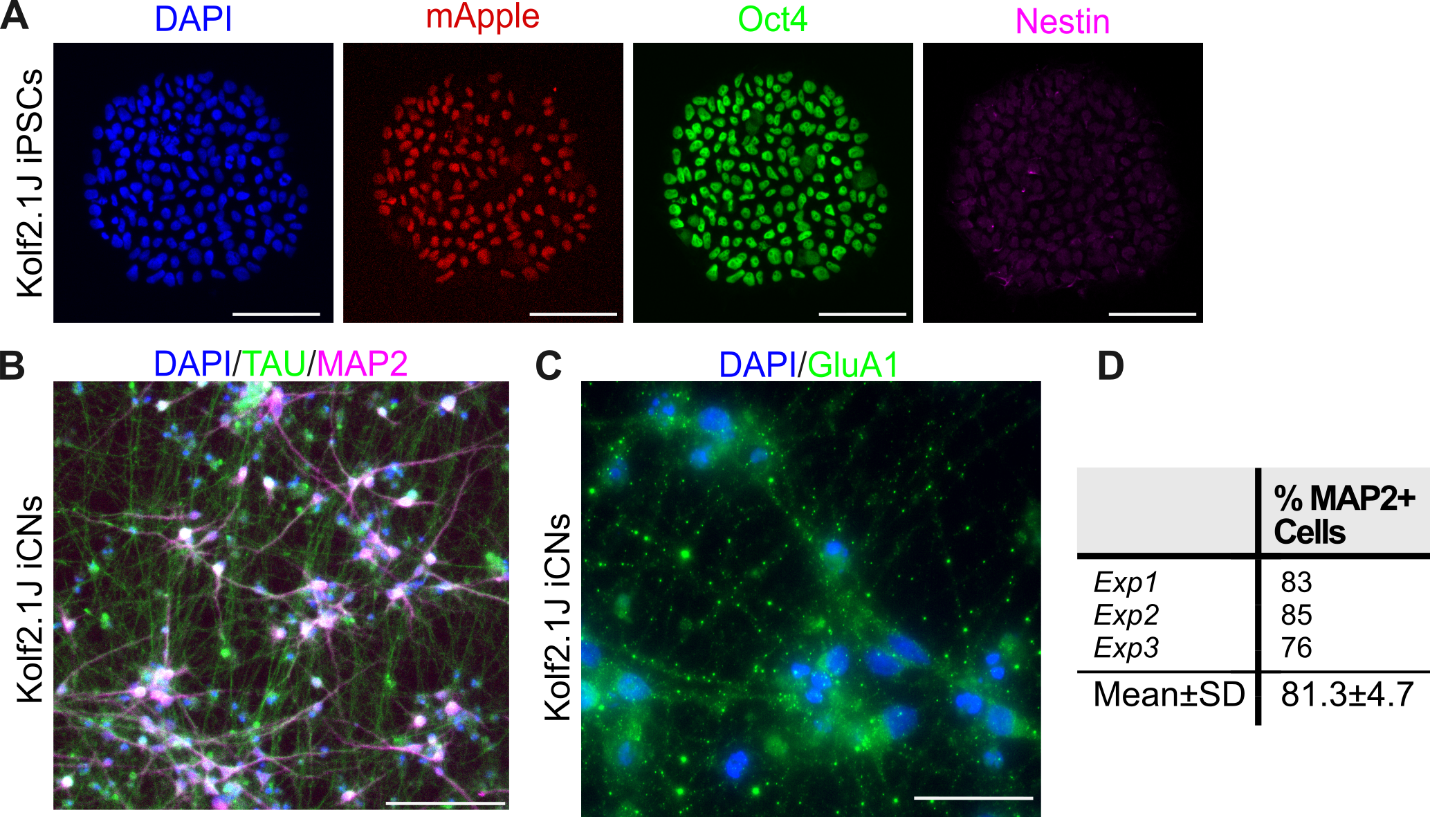


**Supplementary Figure 1. Characterization of Kolf2.1J iPSC-derived cortical neurons.** **A.** Immunocytochemistry of Kolf2.1J iPSCs expressing the pluripotency marker Oct4 (*green*) but not the progenitor marker Nestin (*magenta*). Cells express the red fluorescent protein mApple^NLS^ (*red*), that was integrated in the cell’s genome as part of the differentiation cassette and used as positive selection marker (see *Materials and Methods*). Scale bars 50 µm. **B.** Representative image of differentiated induced cortical neurons (iCNs) stained for MAP2 (*magenta*) and TAU (*green*), confirming neuronal identity. Scale bars are 100µm. **C.** Mature iCNs also express the glutamatergic receptor subunit GluA1 (*green*). Scale bars are 100µm. All nuclei are counterstained with DAPI (*blue*). **D.** Quantification of the efficiency of neuronal differentiation as the percentage of MAP2^+^ cells.


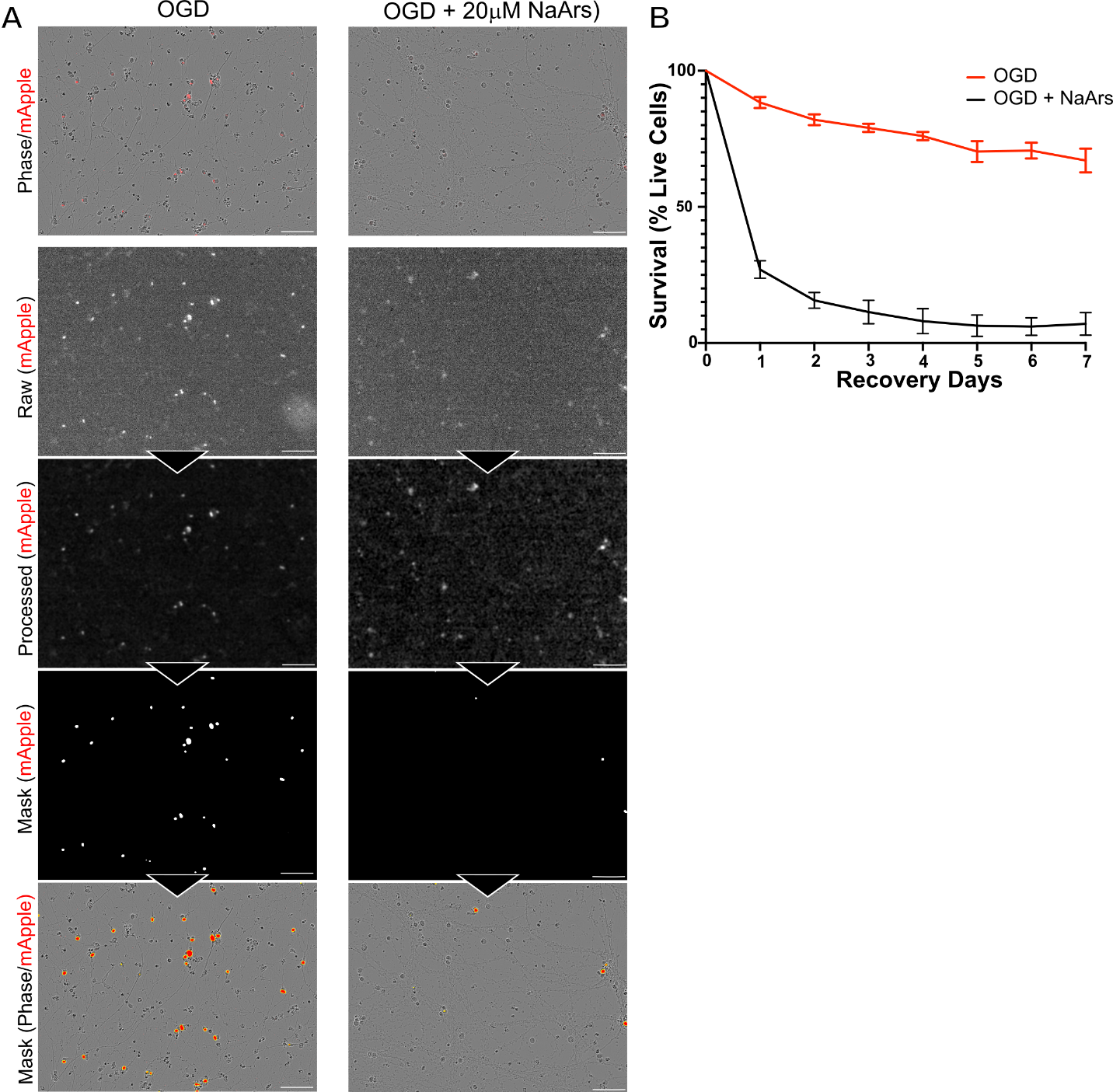


**Supplementary Figure 2. Validation of live-cell screening pipeline using sodium arsenite (NaArs) as toxic agent. A.** Representative images of live cell analysis pipelines in OGD-exposed neurons with or without NaArs, used as a negative control. From top to bottom, columns show phase contrast of neurons with mApple signal (*red*), raw mApple fluorescence, processed mApple signal (ROF denoise, top-hat filter, despeckle, smooth), binary mask of detected cells, and overlay of mask (*red*) with phase contrast. Neurons treated with NaArs (20 µM) exhibited markedly reduced survival compared to untreated controls 7 days post injury. **B.** Quantification of neuronal survival over a 7-day recovery period shows a rapid decline in survival with OGD+NaArs treatment compared to OGD alone. Data are shown as mean ± SEM across independent replicates. Scale bars: 100 µm.


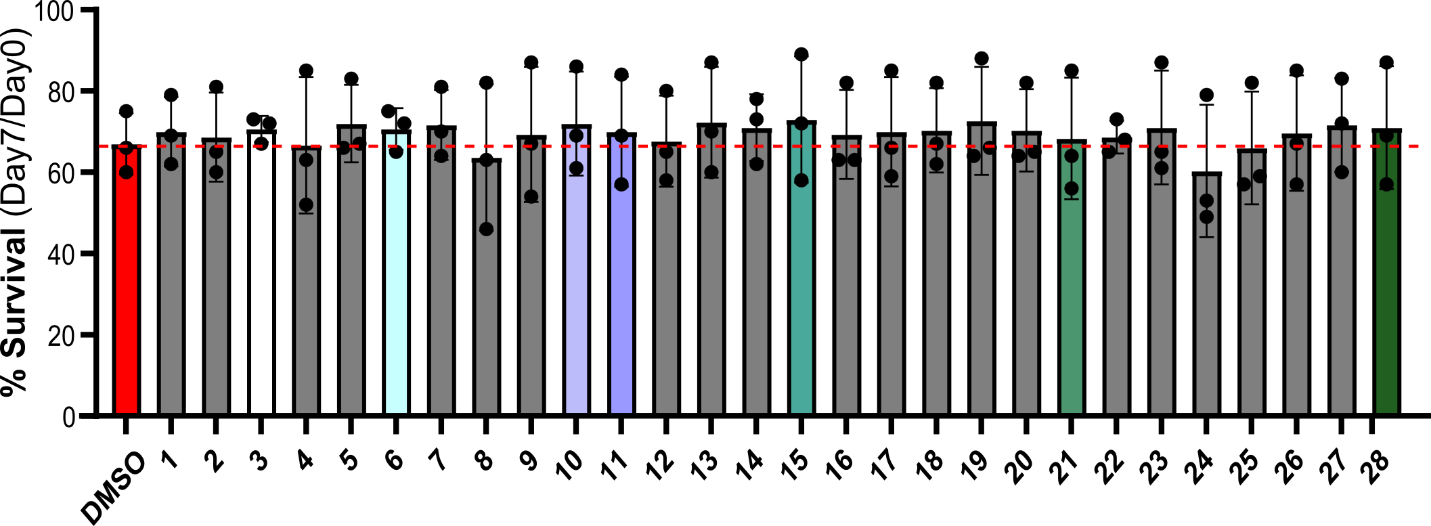


**Supplementary Figure 3. Phytocannabinoids modulate neuronal survival after OGD.** Quantification of survival of iCNs exposed to OGD and treated with DMSO or a panel of 28 phytocannabinoids. Neuronal numbers were evaluated immediately after OGD (Day 0) and after 7 days of recovery. Data is displayed as the percentage of cells still alive at day 7 (n = 3, one way ANOVA with mixed effects model, not significant).


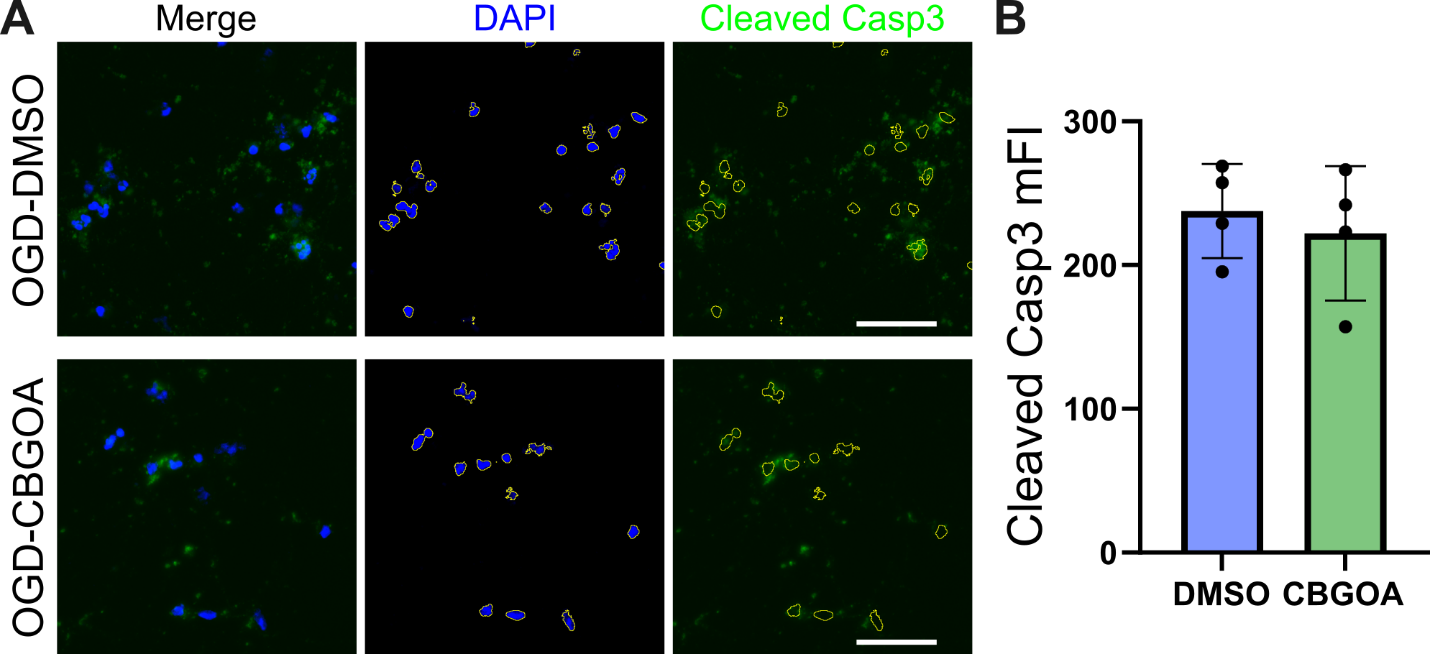


**Supplementary Figure 4.** **CBGOA treatment does not affect caspase 3 activation. A.** Representative images of untreated and CBGOA-treated neurons one day after OGD exposure. Caspase 3 activation was detected using an antibody specific for its cleaved form (Cleaved Casp3, *green*). DAPI (blue) was used to identify cells and define a mask (indicated by the yellow outline) for quantification. **B.** Quantification of cleaved caspase 3 mean fluorescence intensity (mFI) shows no difference between DMSO- and CBGOA-treated neurons at day 1 after OGD (ratio paired two-tailed *t*-test; *n* = 4, p = 0.71).

| **ID Number** | **Compound Name** | **Abbreviation** | **Molecular Weight** |
| --- | --- | --- | --- |
| 1 | cannabidiolic acid methyl ester | CAME | 372.5 |
| 2 | Δ^9^-THCB | Δ^9^-THCB | 300.4 |
| 3 | cannabinol | CBN | 310.43 |
| 4 | cannabinodiol | CBND | 310.44 |
| 5 | cannabicitran | CBT | 314.5 |
| 6 | THCA-A | THCA-A | 358.5 |
| 7 | 11-nor-9-carboxy-9-THC | THCCOOH | 344.4 |
| 8 | cannabidivarin | CBDV | 286.42 |
| 9 | cannabichromene | CBC | 314.46 |
| 10 | 11-hydroxy- Δ^9^-THC | 11H- Δ^9^THC | 330.5 |
| 11 | tetrahydrocannabivarin | THCV | 286.41 |
| 12 | cannabidiolic acid | CBDA | 358.5 |
| 13 | cannabigerovarinic acid | CBGVA | 332.4 |
| 14 | cannabichromevarin | CBCV | 286.4 |
| 15 | cannabigerovarin | CBGV | 288.4 |
| 16 | varinolic acid | Varinolic acid | 196.2 |
| 17 | cannabicyclol | CBL | 314.47 |
| 18 | Δ^8^-THCA-A | Δ^8^-THCA-A | 358.5 |
| 19 | cannabinol monomethyl ether | CBGM | 324.5 |
| 20 | cannabivarin | CBV | 282.4 |
| 21 | cannabigerorcinic acid | CBGOA | 304.4 |
| 22 | 6α-hydroxy cannabidiol | 6H-CBD | 330.5 |
| 23 | cannabigerol | CBG | 316.48 |
| 24 | cannabigerolic acid | CBGA | 360.49 |
| 25 | cannabidibutol | CBDB | 300.4 |
| 26 | cannabidiphorol | CBDP | 342.5 |
| 27 | Δ^9^-THCP | Δ^9^-THCP | 342.5 |
| 28 | cannabidiol | CBD | 314.47 |

**Supplementary Table 1. List of the tested phytocannabinoids.** Compound ID number used throughout the study is shown, together with full name, abbreviation, and molecular weight.
